# Factors Affecting the Effectiveness of Inorganic Silicate Sealer Material through Multi-Quality Characteristics

**DOI:** 10.3390/ma6031191

**Published:** 2013-03-22

**Authors:** Si-Yu Zou, Ran Huang, Mao-Chieh Chi, Hui-Mi Hsu

**Affiliations:** 1 Department of Harbor and River Engineering, National Taiwan Ocean University, 2 Pei-Ning Road, Keelung, 20224, Taiwan; E-Mail: siyuzou@gmail.com; 2 Department of Fire Science, WuFeng University, 117, Sec 2, Chiankuo Road, Minhsiung, Chiayi 62153, Taiwan; E-Mail: jackchi@wfu.edu.tw; 3 Department of Civil Engineering, National Ilan University, No.1, Sec. 1, Shen-Lung Road, I-Lan 26047, Taiwan; E-Mail: hmhsu@niu.edu.tw

**Keywords:** Taguchi method, grey relational analysis, multi-quality characteristics, inorganic silicate sealer material

## Abstract

This study investigates the effectiveness of concrete protection with two inorganic silicate sealer materials (ISSMs). The Taguchi method and grey relational analysis (GRA) have been used to identify the key factors influencing concrete protection provided by the surface treatment. Seven control factors with two levels were selected. By using the orthogonal array L_12_ (2^7^), 12 experiments are chosen and four tests—the compressive strength test, resistivity test, absorption test and permeability test—were performed. Results have shown that the major factors affecting the protection effectiveness of ISSM are the water-binder ratio of mortar substrate, age of substrate for sealer application, addition of pozzolanic material and sealer type.

## 1. Introduction

Concrete has been one of the most widely used materials in construction for a long time. However, physical and chemical factors often lead to the degradation of concrete during its life cycle [1,2,3]. Concrete is a porous and heterogeneous composite material. The pores may accumulate due to leaching of Ca(OH)_2_, one of the hydration products [4,5]. Increasing pores enable the transport of harmful substances through the capillary pores from external exposed environment [6,7], which would damage the internal structure and thus reduce strength and durability of concrete.

To modify the pore structure of concrete, pozzolanic materials could be added to produce secondary hydration [8,9]. Thus, total pore volume decreases to achieve denser, stronger and durable concrete. Surface coating materials are frequently applied to the surface of concrete to form a protective layer, block pores or to decrease pore size. Consequently, harmful substances are prevented from entering through concrete cracks or pores.

Silicate sealer primarily composed of cement can be used as a concrete surface protective material [10]. In humid condition, silicate sealers would permeate into concrete through capillary pores and react with Ca(OH)_2_ to produce insoluble crystals [11], which would block the transporting path. Several inorganic silicate sealer materials have penetration characteristics it is essential to consider the penetration depth of sealer as a factor affecting sealer effectiveness [12]. Penetration depth is related to the type of sealer, substrate concrete and the exposed environment [13].

The assessment of an optimal mixture for desired quality is an important issue in the field of material engineering. Therefore, the design of experiments (DOE) is critical to identify the quality and characteristics of materials within limited time and cost. Utilization of Taguchi method is to minimize the experiment number using standard orthogonal array and signal/noise ratio [14]. Usually it can only consider single quality characteristics. On the other hand, Grey Relational Analysis (GRA) is usually employed to deal with multi-quality characteristics. Therefore, combining these two methods could enhance quality and reliability [15,16,17].

To determine the effectiveness of multi-quality characteristics in ISSM, this study adopted the sealer materials used in previous studies [10,18,19,20] as the control factors in experiments. Meanwhile, the Taguchi method and GRA were employed in three-phase analysis. Firstly, this study used Taguchi’s orthogonal array to evaluate the influence of the control factors, quantify the protective effects of sealer materials on concrete protection, and identify the important factors influencing quality characteristics. Secondly, all normalizing experiment variables ranked the grey relational grades of multi-quality characteristics. Finally, this study integrated the Taguchi method and the entropy weights by using GRA to establish entropy weight-based grey relational values. These values were then used to conduct an analysis of variance (ANOVA) and derive a factor response graph for multi-quality characteristics in order to identify the important factors associated with individual and multi-quality characteristics.

## 2. Experimental Procedure

### 2.1. Materials and Specimens

**Figure 1:**
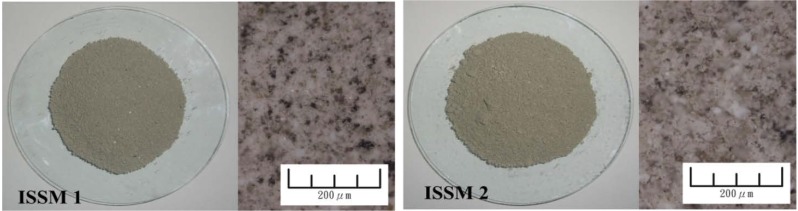
Inorganic Silicate Sealer Material.

Two different ISSMs were used in the experiment. Both ISSMs mainly composed of cement-based materials are similar grey powders as shown in Figure 1. The chemical compositions of cement, fly ash, ISSM1 and ISSM2 are listed in Table 1. Type I Portland cement with a specific gravity of 3.15 and class F fly ash for partial cement replacement (by weight) were used. River sand is used as fine aggregate with specific gravity, water absorption, and fineness modulus as 2.56, 1.85%, and 2.82, respectively. Four mixtures were designed and the mix proportions of mortar were listed in Table 2. ISSM1 and ISSM2 were firstly mixed with water (the amount of the sealer material was proportional to water by mass) before applying to the surface of cubic and cylindrical mortar specimens (substrate).

**Table 1:** Chemical compositions of ISSM, cement, and fly ash.

Chemical compositions (%)	Substrate		Inorganic silicate sealer material	
	cement	fly ash	ISSM1	ISSM2
Calcium oxide, CaO	63.56	2.82	25.23	34.33
Silicon dioxide, SiO_2_	21.04	56.48	33.77	28.46
Aluminum oxide, Al_2_O_3_	5.46	20.34	5.90	4.47
Ferric oxide, Fe_2_O_3_	2.98	6.61	5.27	4.75
Sulfur trioxide, SO_3_	2.01	0.25	1.07	1.38
Sodium oxide, Na_2_O	0.32	0.33	15.00	12.12
Potassium oxide, K_2_O	0.70	0.80	0.83	1.10
Magnesium oxide, MgO	2.52	0.93	6.20	7.43
Loss on ignition, L.O.I.	1.38	2.76	1.54	1.84
Others	0.03	8.68	5.19	4.12

**Table 2:** Mix proportions of mortar

Mixture	Mix proportions of mortar (kg/m^3^)				
	W/C ratio	Cement	Water	Fly ash	Sand
Mix 1	0.45	523	235	0	1437
Mix 2	0.45	413	233	103	1421
Mix 3	0.65	471	306	0	1295
Mix 4	0.65	373	303	93	1282

### 2.2. Experiments

ISSM was employed by mixing the sealer material with water (the amount of the sealer material was proportional to water by mass) and then coated uniformly with the mixture onto the surface of the cement mortar. The specimens were cured in moist curing at relative humidity of 95% RH and the temperature of 20 ± 2 °C and air curing at relative humidity of 70% RH and the temperature of 23 ± 4 °C until testing, respectively. Compressive strength test of the specimens was conducted according to ASTM C39M-12. Water absorption was made in accordance with ASTM C642. For each mixture, 50 × 50 × 50 mm cubes were prepared. Permeability was measured using CRD48-96 standard test methods, and cylinders of size 100 mm diameter and 50 mm height were cast. In addition, the cylindrical specimens (φ 100 × 300 mm) were used for the concrete resistivity measurement using four-point Wenner array probe technique. All specimens were tested in triplicate sets for each mixture at the age of 28 days.

### 2.3. Taguchi Method

Taguchi method is a design and analysis of experiments for the purpose of designing and improving quality. Taguchi method primarily involves orthogonal arrays and Signal to Noise ratio (S/N). Orthogonal arrays can reduce the number of experiments, and thus, overall costs. On the analysis side, Taguchi advocated the S/N ratio as a single indicator that jointly and simultaneously considers the average value and standard deviation of test results to determine the relative importance of the factors under study [15,21]. In the Taguchi method, S/N ratios and factor response graphs and tables are firstly derived from the experimental results to understand the influence trends associated with the factors at different levels. ANOVA is then performed to determine the degree of influence of each control factor as well as the contribution of each factor to the experiment.

#### 2.3.1. Quality Characteristics

In Taguchi method, S/N ratio is used to measure the deviation of the quality characteristics from the desired value. The S/N ratio for each process parameter is calculated based on S/N analysis function. A larger S/N ratio is normally consistent with better quality characteristics. Quality characteristics can be divided into larger-the-better (LTB), smaller-the-better (STB), and nominal-the-best (NTB). This study set compressive strength and resistivity as an LTB characteristic Equation (1) and water absorption and permeability as STB characteristics Equation (2) in the investigation of single quality characteristics.

(1) LTB S/N ratio

This is applicable for quality characteristics that are better when bigger.

(1) $$SN_{LTB}=-10\text{log}\left(\frac{\sum_{i=1}^{n}\frac{1}{y_{i}^{2}}}{n}\right)$$

(2) STB S/N ratio

This is applicable for quality characteristics that are better when smaller.

(2) $$SN_{STB}=-10{\text{log}}_{10}\left({\bar{y}}^{2}+s_{n}^{2}\right)$$

#### 2.3.2. Orthogonal Array

This study identified the key factors influencing the protective effects of ISSM for the treatment of concrete surfaces. Orthogonal arrays can reduce the number of experiments, and thus, overall costs. For instance, a full factorial experiments with seven control factors and two levels would produce a total of n = 2^7^ = 128 sets of results, among which an optimal design would have to be determined. However, this approach is extremely inefficient. In contrast, Taguchi’s L_12_ (2^7^) orthogonal array would require only 12 experiments. Seven control factors and two levels were chosen and L_12_ (2^7^) orthogonal array was used. The designation of control factors and factor levels are listed in Table 3. Orthogonal array design of L_12_ (2^7^) is shown in Table 4. Responses to the factors were then analyzed.

**Table 3:** Designation of control factors and factor levels.

Control factors		Level	
A	Sealer Materials type	ISSM1	ISSM2
B	Sealer Materials ratio	5:2	5:3
C	Sealer layers	1	2
D	Sealer cumulative time (days)	28	3
E	Water-binder ratio of substrate	0.45	0.65
F	Moist curing age (days)	3	28
G	Adding fly ash in substrate	0%	20%

**Table 4:** L_12_ (2^7^) Orthogonal array.

Experiment No.	Control factors and levels						
	A	B	C	D	E	F	G
1	1	1	1	1	1	1	1
2	1	1	1	1	1	2	2
3	1	1	2	2	2	1	1
4	1	2	1	2	2	1	2
5	1	2	2	1	2	2	1
6	1	2	2	2	1	2	2
7	2	1	2	2	1	1	2
8	2	1	2	1	2	2	2
9	2	1	1	2	2	2	1
10	2	2	2	1	1	1	1
11	2	2	1	2	1	2	1
12	2	2	1	1	2	1	2

#### 2.3.3. Analysis of Variance

The use of S/N ratios provides only the degree of influence that the factor levels exert on the experiment. However, ANOVA enables a further evaluation of the contribution of each factor and determines its importance. In this study, ANOVA and F-test were performed to see statistically significant process control factors and percent contribution of experimental. Firstly, the S/N ratio sum of squares for each factor is given by Equation (3). Then the between-group variability can be calculated by Equation (4). Finally, F-test and contribution ratio can be simplified to Equations (5) and (6), respectively. All equations are described as following:

S/N ratio sum of squares

(3) $$SS_{factor}=n\times \sum_{i=1}^{n}{\left(x_{i}-\bar{x}\right)}^{2}$$

Variance between groups

(4) $$Var=\frac{SS_{factor}}{d.o.f}$$

where *d.o.f.* is degree of freedom.

F-test

(5) $$F=\frac{Var}{Var_{e}}$$

where Var_e_ is the within-group variability.

Contribution ratio

(6) $$P\%=\frac{SS_{factor}}{SS_{total}}\times 100\%$$

where SS_total_ is the sum of SS_factor_

### 2.4. Grey Relational Analysis (GRA)

Grey Relational Analysis (GRA) a normalization evaluation technique is extended to solve the complicated multi-performance characteristics optimization effectively. The grey theory is based on the random uncertainty of small samples which developed into an evaluation technique to solve certain problems of system that are complex and having incomplete information. By investigating a small amount of data and multiple characteristics, reference series can be derived from different series of data. Deriving the overall relation grade of the series or the relational grades of the individual series factor changes can also integrate the data of multiple independent quality characteristics into a single relational order of optimal multi-quality characteristics. This prevents overlooking many quality characteristics for the sake of one [16,22]. The results can be compared with the order obtained using Taguchi methods.

In GRA, calculating the results from L_12_ (2^7^) orthogonal array data to generate grey relations Equations (7) and (8), referential sequences, grey relational coefficients Equation (9), and grey relational grades Equations (10) and (11) must be done firstly in this study. In this manner, it was possible to obtain equally weighted, entropy weight-based grey relational orders, of which the former involves equally distributing the grey relational coefficients. The weights of the grey relational grades are determined using entropy. A higher standard deviation in the quality characteristic results leads to a higher entropy weight. This reveals the distribution of the weight values, and differences in the grey relational orders can be used to supplement the preliminary evaluations derived using Taguchi method.

For the form “the-higher-the-better”, the original sequence can be normalized as

(7) $$x_{i}(k)=\frac{x_{i}(k)-\text{min}.x_{i}(k)}{\text{max}.x_{i}(k)-\text{min}.x_{i}(k)}$$

For the form “the-smaller-the-better”, the original sequence can be normalized as

(8) $$x_{i}(k)=\frac{\text{max}.x_{i}(k)-x_{i}(k)}{\text{max}.x_{i}(k)-\text{min}.x_{i}(k)}$$

Grey relational coefficients

(9) $$\gamma \left(x_{0}\left(k\right),x_{i}\left(k\right)\right)=\frac{\Delta \text{min}+\zeta \cdot \Delta \text{max}}{\Delta_{0,i}(k)+\zeta \cdot \Delta \text{max}}$$

Equally weighted grey relational grades

(10) $$\gamma (x_{i},x_{j})=\frac{1}{n}\sum_{k=1}^{n}\gamma \left(x_{0}(k),x_{i}(k)\right)$$

Entropy weighted grey relational grades

(11) $$\gamma (x_{i},x_{j})=\sum_{k=1}^{n}\beta_{k}\gamma \left(x_{i}(k),x_{j}(k)\right)$$

## 3. Results and Discussion

### 3.1. S/N Ratios Calculations of Taguchi Method

**Figure 2:**
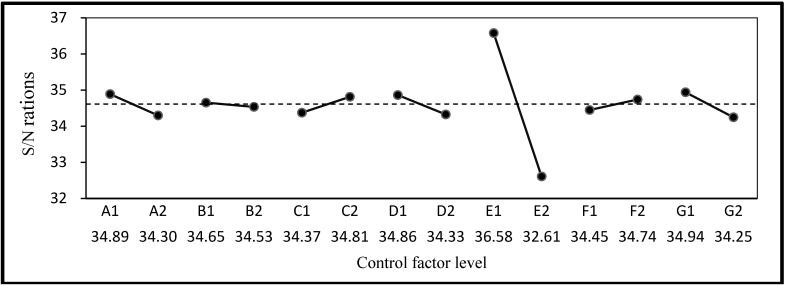
S/N ratio response graph for compressive strength.

**Figure 3:**
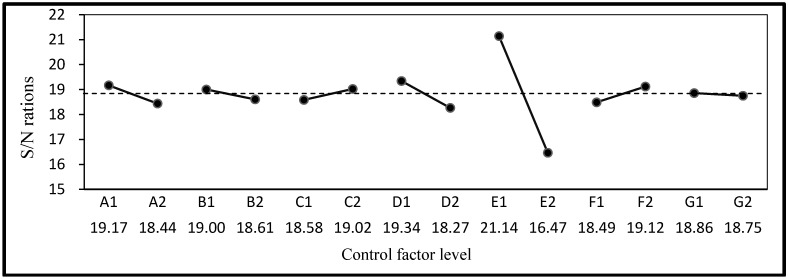
S/N ratio response graph for Resistivity.

**Figure 4:**
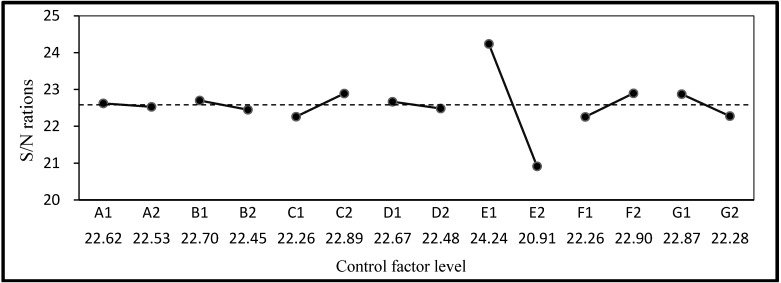
S/N ratio response graph for water absorption.

**Figure 5:**
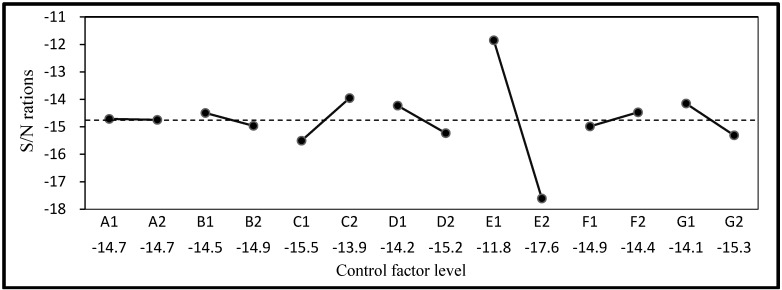
S/N ratio response graph for permeability.

The mean, standard deviation, and S/N ratio of the compressive strength, resistivity, water absorption, and permeability are listed in Table 5. Compressive strength and resistivity were set as LTB characteristic Equation (1) and water absorption and permeability as STB characteristic Equation (2). The S/N ratios and factor response graphs derived according to quality characteristics are shown in Figure 2, Figure 3, Figure 4 and Figure 5. It can be seen that the factor responses of E (water-binder ratio of substrate) were relatively greater than any others. A larger change in the response values indicates a higher degree of influence. It indicates that water-binder ratio of substrate has a significant influence on protection effectiveness of ISSM.

As listed in Table 5, experiment No.1 has the highest S/N ratio of the twelve experiments regarding the LTB characteristic, with 37.10 dB of the compressive strength and 22.04 dB of the resistivity. It indicates that the experiment No.1 has the superior performance on compressive strength and resistivity. Thus, experiment No.1 can be considered as the optimal designation of mixture. In contrast, experiment No.10 delivered the optimal effect with 24.45 dB of water absorption rate and −10.19 dB of permeability as to the STB characteristic. It shows that experiment No.10 has the lower water absorption and permeability than any others. Based on these four experimental values, it is found that it is not easy to effectively determine the key protective factors. Therefore, a GRA was employed to weigh and verify the key factors.

**Table 5:** Experimental data and calculated S/N ratios.

No.	Compressive strength			Resistivity			Water absorption			Permeability		
	Ave	S	S/N (dB)	Ave	S	S/N (dB)	Ave	S	S/N (dB)	Ave	S	S/N (dB)
	(MPa)			mV			(%)			(g)		
1	71.63	1.49	37.10	12.67	0.50	22.04	6.02	0.0023	24.40	3.75	0.30	−11.49
2	69.73	2.12	36.86	12.60	0.17	22.01	6.33	0.0016	23.97	4.07	0.61	−12.26
3	46.94	1.51	33.42	6.37	0.12	16.08	8.84	0.0022	21.07	6.71	1.78	−16.73
4	40.25	2.17	32.07	6.83	0.32	16.67	9.91	0.0020	20.08	9.08	0.54	−19.17
5	47.68	1.07	33.56	7.53	0.35	17.52	8.09	0.0017	21.84	6.34	1.06	−16.12
6	65.44	1.12	36.31	10.87	0.60	20.70	6.04	0.0011	24.38	4.22	0.14	−12.51
7	59.94	2.43	35.54	10.07	0.38	20.05	6.38	0.0023	23.90	4.25	0.49	−12.60
8	45.15	1.26	33.09	8.33	0.40	18.40	8.21	0.0025	21.71	5.99	0.57	−15.58
9	39.39	0.72	31.90	5.93	0.32	15.44	8.75	0.0038	21.15	8.21	0.83	−18.32
10	70.49	2.66	36.95	11.77	0.31	21.41	5.99	0.0016	24.45	3.21	0.46	−10.19
11	68.42	1.50	36.70	10.80	0.36	20.66	6.08	0.0012	24.33	3.97	0.66	−12.05
12	38.10	1.41	31.61	5.43	0.25	14.68	10.42	0.0052	19.64	9.68	0.86	−19.74

### 3.2. Analysis of Variance

In this study, seven control factors are regrouped into three phases: coating materials (A, B and C factors), curing conditions (D and F factors) and based materials (E and G factors). The contributions of the control factors to the experiment are listed in Table 6. It is shown that factor E (water-binder ratio of substrate) has the highest contribution ratio in the seven factors, with 90.02% of the compressive strength, 83.98% of the resistivity, 88.77% of the water absorption rate, and 84.19% of the permeability, respectively. In the phase of coating materials, factor A (Sealer Materials type) has a significant influence on compressive strength and resistivity, which factor C (Sealer layers) on water absorption and permeability. As to the phase of curing condition, factor D has the higher effect on the properties of specimens compared to factor F.

**Table 6:** Results of ANOVA for contribution ratio.

Control factor	Compressive strength	Resistivity	Water absorption	Permeability
A	1.99%	2.05%	0.07%	0.00%
B	0.08%	0.60%	0.51%	0.56%
C	1.10%	0.74%	3.18%	6.11%
D	1.64%	4.45%	0.27%	2.55%
E	90.02%	83.98%	88.77%	84.19%
F	0.48%	1.53%	3.30%	0.67%
G	2.74%	0.04%	2.85%	3.41%
Error	1.94%	6.61%	1.05%	2.52%
Total	100%	100%	100%	100%

### 3.3. Grey Relational Analysis

In the GRA, the quality characteristics (compressive strength, resistivity, water absorption, and permeability results) were converted into a single optimal order combination. The units and values of the quality characteristics in the analysis varied; therefore, grey relational generation was performed through Equations (7) and (8) to normalize the corresponding values between 0 and 1. Table 7 presents the grey relational coefficients Equation (9) and grey relational orders in which the grey relational grades were divided into equally weighted grades Equation (10) and entropy weight-based grades Equation (11) prior to ranking. As the name suggests, equal weights indicate that the weight values were equally distributed among the quality characteristics. Entropy weights, on the other hand, were based on the differences in data related to quality characteristics. The entropy weights for compressive strength, resistivity, water absorption, and permeability were 0.3055, 0.2689, 0.2031, and 0.2224, respectively. The higher entropy weight for compressive strength was caused by the greater differences in the experimental data.

**Table 7:** Grey relational coefficients and grey relational grades.

No.	Grey relational coefficients				Grey relational grades			
	Compressive strength	Resistivity	Water absorption	Permeability	Equally weighted	Rank	Entropy weights	Rank
1	1.0000	1.0000	0.9814	0.7858	0.9418	2	0.9486	1
2	0.9205	0.9909	0.8339	0.6979	0.8608	3	0.8723	3
3	0.4275	0.3815	0.4161	0.4220	0.4118	9	0.4116	9
4	0.3532	0.4067	0.3551	0.3471	0.3655	11	0.3666	10
5	0.4371	0.4487	0.4795	0.4461	0.4529	8	0.4509	8
6	0.7780	0.7324	0.9713	0.6731	0.7887	5	0.7817	5
7	0.6381	0.6485	0.8128	0.6647	0.6910	6	0.6823	6
8	0.4063	0.5023	0.4678	0.4698	0.4615	7	0.4587	7
9	0.3458	0.3579	0.4221	0.3700	0.3740	10	0.3700	11
10	0.9490	0.8533	1.0000	1.0000	0.9506	1	0.9450	2
11	0.8732	0.7271	0.9511	0.7192	0.8176	4	0.8155	4
12	0.3333	0.3333	0.3333	0.3333	0.3333	12	0.3333	12

The ranking in grey relational grades of multi-quality characteristics is listed in Table 7. It can be seen that these two types of ranking methods are slightly different because of contrasting weight calculations. The optimal results from the equal weights occurred in the tenth ranking of the grey relational grades, while for entropy weights the optimal results occurred in the first ranking. These results were primarily attributed to the higher weights in the entropy weights because of their compressive strength and resistivity, resulting in a discrepancy in their ranking.

### 3.4. The Weighted Grey-Taguchi Method

**Figure 6:**
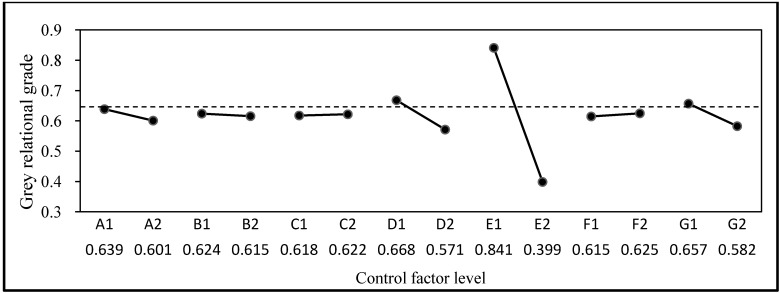
Grey relational grade responses graph for multi-quality factors.

The framework of the weighted Grey-Taguchi method is based on the ANOVA and response graphs of the Taguchi method. This study adopted the entropy weight-based grey relational grades of GRA and the Taguchi method provided a comprehensive quantified presentation. ANOVA for the weighted Grey-Taguchi method and the grey relational grade response graph for multi-quality factors are displayed in Table 8 and Figure 6.

Regarding the multi-quality factor response, a comprehensive assessment suggested that, for concrete substrates, factor E (*i.e.*, water-binder ratio of the substrate) and factor G (*i.e.*, the added pozzolanic material) have the most contribution of 89.99% and 2.55%, respectively, while for the ISSM, factor A (*i.e.*, the sealer materials type) and factor D (*i.e.*, the sealer cumulative time) have the most contribution of 4.31% and 0.66%, respectively.

**Table 8:** Results of ANOVA for weighted Grey-Taguchi method.

Control factor	Sum of squares	Degrees of freedom	Variance	F-test	Contribution ratio
A	0.004	1	0.004	1.092	0.66%
B	0.000	1	0.000	0.054	0.03%
C	0.000	1	0.000	0.012	0.01%
D	0.028	1	0.028	7.164	4.31%
E	0.587	1	0.587	149.439	89.99%
F	0.000	1	0.000	0.080	0.05%
G	0.017	1	0.017	4.228	2.55%
Error	0.016	4	0.004	-	2.41%
Total	0.652	11	-	-	100.00%

### 3.5. Investigation of Key Protective Factors

ISSM employs a protective mechanism of permeable crystalline. The penetration depth of the sealer material is crucial to be considered. Furthermore, the penetration depth is determined by the properties of the sealer material, substrate, and the environment. This study adopted the results from the weighted Grey-Taguchi method as the basis for conducting the statistical analysis and discussions on the key factors. Regarding the substrate, factor E (*i.e.*, water-binder ratio of the substrate) and factor G (*i.e.*, the added pozzolan material) were identified as the key factors in protective effectiveness because of the large discrepancy in the level response caused by the densification of the substrate itself. Comparatively, the protective effect of the sealer material was not the primary cause. The protective mechanism of the ISSM entails, in water, the silicate sealer permeating the concrete structure through capillary pores, producing a chemical reaction with the concrete’s free Ca(OH)_2_, forming an insoluble crystalline. The greater the penetration depth, the more evident the enhancement of the porosity. Consequently, the greater the substrate densification, the less evident the effect of sealing sing the ISSM. This is because the porosity and connectivity of the specimen cannot provide the conditions for permeation. In contrast, the effect of improved protection is more evident when sealing specimens with greater porosity and higher connectivity.

Regarding the sealing material, factor A (*i.e.*, the sealer materials type) and factor D (*i.e.*, the sealer cumulative time) were identified as the key factors. As shown in Equation (12), the sodium silicate in the ISSM protective mechanism reacted with water and the Ca(OH)_2_ from the hydration products to form Calcium-Silicate-Hydrate (C-S-H), a needle-shaped substance that fills the pores [23]. The proportion of the sodium silicate in the composition or formula should be regarded as a critical factor that can help crystal growth within the specimen. In addition, as proposed by previous studies [11], this type of sealing material requires cumulative time to perform internal chemical reactions to achieve higher protective quality, which corresponds to the results that suggest that factor D (*i.e.*, the sealer cumulative time) is a key factor.

Na_2_SiO_3_ + yH_2_O + xCa(OH)_2_→xCaO•SiO_2_•yH_2_O + 2NaOH [19] (12)

## 4. Conclusions

This study employed the Taguchi method and GRA to identify the key factors influencing the concrete protection provided by the surface treatment of reinforced concrete structures. The main conclusions extracted are following:

1. The weighted Grey-Taguchi method quantifies the performance of multi-quality characteristics, thus providing assistance in the search for key factors for ISSM in concrete protection.
2. Regarding the contribution percentage of the multi-quality characteristics, factors A, D, E, and G showed the higher contribution percentages than any others. Factors A and D affect the protective property of the specimen quality because of the property of the sealer material. In contrast, the effects of factors E and G are caused by the quality of the substrate.
3. The optimal results for the parameters of the multi-quality characteristics are obtained by using GRA. The optimal results from the equal weights occur in the tenth ranking of the grey relational grades, while for entropy weights the optimal results occur in the first ranking.
4. The Standard deviation in experimental data is a key element influencing factor. A higher discrete degree in standard deviation affects the importance of individual influence factors as well as the entropy weights of grey relational grades.
5. The ISSM1(A1), sealer materials ratio of 5:2(B1), and 2 sealer layers(C2) have the superior performance for the surface protection of concrete in coating materials, whereas sealer cumulative time 28days(D1) and moist curing age 28days(F2) in environmental conditions. In addition, the less effectiveness of concrete protection results in the more density of concrete.
